# Improving crop yield potential: Underlying biological processes and future prospects

**DOI:** 10.1002/fes3.435

**Published:** 2022-12-02

**Authors:** Alexandra J. Burgess, Céline Masclaux‐Daubresse, Günter Strittmatter, Andreas P. M. Weber, Samuel Harry Taylor, Jeremy Harbinson, Xinyou Yin, Stephen Long, Matthew J. Paul, Peter Westhoff, Francesco Loreto, Aldo Ceriotti, Vandasue L. R. Saltenis, Mathias Pribil, Philippe Nacry, Lars B. Scharff, Poul Erik Jensen, Bertrand Muller, Jean‐Pierre Cohan, John Foulkes, Peter Rogowsky, Philippe Debaeke, Christian Meyer, Hilde Nelissen, Dirk Inzé, René Klein Lankhorst, Martin A. J. Parry, Erik H. Murchie, Alexandra Baekelandt

**Affiliations:** ^1^ School of Biosciences University of Nottingham, Sutton Bonington campus Loughborough UK; ^2^ Université Paris‐Saclay, INRAE, AgroParisTech Institut Jean‐Pierre Bourgin (IJPB) Versailles France; ^3^ Institute of Plant Biochemistry, Cluster of Excellence on Plant Sciences (CEPLAS) Heinrich‐Heine‐Universität Düsseldorf Düsseldorf Germany; ^4^ Lancaster Environment Centre Lancaster University Lancaster UK; ^5^ Laboratory for Biophysics Wageningen University and Research Wageningen The Netherlands; ^6^ Centre for Crop Systems Analysis, Department of Plant Sciences Wageningen University & Research Wageningen The Netherlands; ^7^ Plant Biology and Crop Sciences University of Illinois at Urbana‐Champaign Urbana Illinois USA; ^8^ Plant Sciences Rothamsted Research Harpenden UK; ^9^ Department of Biology, Agriculture and Food Sciences, National Research Council of Italy (CNR), Rome, Italy and University of Naples Federico II Napoli Italy; ^10^ Institute of Agricultural Biology and Biotechnology National Research Council (CNR) Milan Italy; ^11^ Copenhagen Plant Science Centre, Department of Plant and Environmental Sciences University of Copenhagen Copenhagen Denmark; ^12^ BPMP, Univ Montpellier, INRAE, CNRS Institut Agro Montpellier France; ^13^ Department of Food Science University of Copenhagen Copenhagen Denmark; ^14^ Université de Montpellier ‐ LEPSE – INRAE Institut Agro Montpellier France; ^15^ ARVALIS‐Institut du végétal Loireauxence France; ^16^ INRAE UMR Plant Reproduction and Development Lyon France; ^17^ Toulouse University INRAE, UMR AGIR Toulouse France; ^18^ IJPB UMR1318 INRAE‐AgroParisTech‐Université Paris Saclay Versailles France; ^19^ Department of Plant Biotechnology and Bioinformatics Ghent University Ghent Belgium; ^20^ VIB Center for Plant Systems Biology Ghent Belgium; ^21^ Wageningen Plant Research Wageningen University & Research Wageningen The Netherlands

**Keywords:** crop improvement, crop yield, food supply, nutrient remobilisation, organ growth, photosynthesis

## Abstract

The growing world population and global increases in the standard of living both result in an increasing demand for food, feed and other plant‐derived products. In the coming years, plant‐based research will be among the major drivers ensuring food security and the expansion of the bio‐based economy. Crop productivity is determined by several factors, including the available physical and agricultural resources, crop management, and the resource use efficiency, quality and intrinsic yield potential of the chosen crop. This review focuses on intrinsic yield potential, since understanding its determinants and their biological basis will allow to maximize the plant's potential in food and energy production. Yield potential is determined by a variety of complex traits that integrate strictly regulated processes and their underlying gene regulatory networks. Due to this inherent complexity, numerous potential targets have been identified that could be exploited to increase crop yield. These encompass diverse metabolic and physical processes at the cellular, organ and canopy level. We present an overview of some of the distinct biological processes considered to be crucial for yield determination that could further be exploited to improve future crop productivity.

## Graphical abstract

The manuscript is part of a review collection from the CropBooster‐P project (https://www.cropbooster‐p.eu/). In this review, we present an overview of some of the distinct biological processes considered to be crucial for yield determination and recent updates in the respective fields. In the future, these could further be exploited to improve crop productivity.

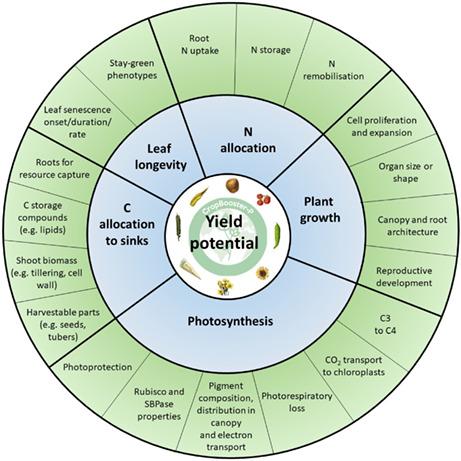

## 1 INTRODUCTION

Based on projections of global population growth, 9.7 billion people will need to be sustainably fed by 2050. Economic growth will enrich this population, which will likely lead to increased overall food consumption. Besides the increased demand for food and animal feed, there will also be an increasing pressure arising from competing uses for agricultural products, for example to allow a transition from a fossil‐based towards a bio‐based economy and to limit global climate change through sustainable energy supplies (Clark et al., 2020). Moreover, crop production will need to be increased using the same or even reduced land area to allow for more biodiversity by returning agricultural land to its natural state. Crop production, however, will be challenged by climate change, including changes in temperature and precipitation, and by an increased incidence of extreme weather events, which all decrease yield stability. Total agricultural productivity has been estimated to being reduced by 21% since 1961 due to climate change (Ortiz‐Bobea et al., 2021).

The future requirements for our crops are undeniably diverse and highly demanding. In the coming decades, one of humanity's greatest challenges will be to sustainably improve crop nutritional quality (Scharff et al., 2021) and yield. Here, yield refers to the total amount of crop biomass produced per unit area per year (Zhu et al., 2010). Increasing yield will depend on selecting the best traits, technologies and crops for breeding and crop management of plants, targeting sustainable increases in total productive potential.

**FIGURE 1:**
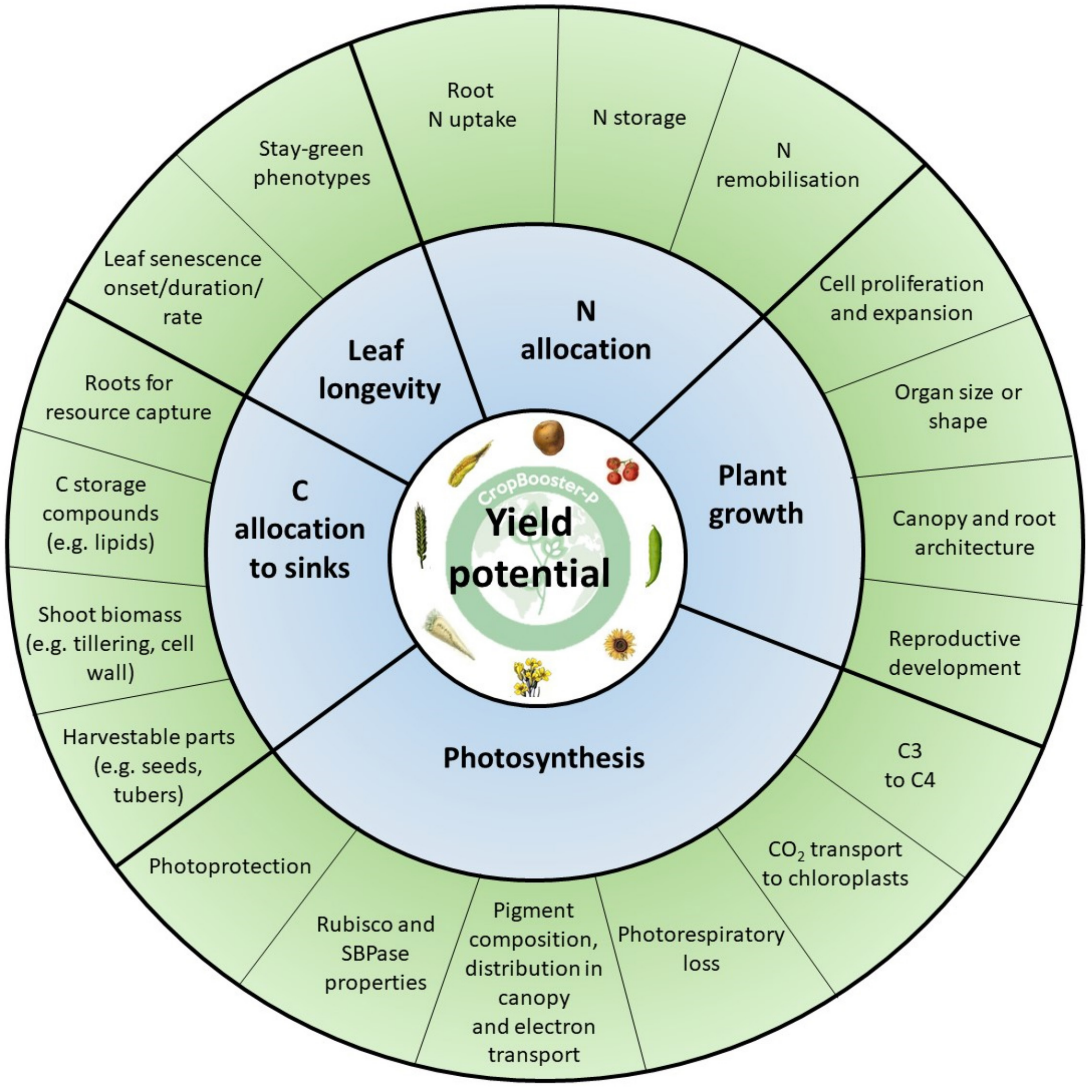
Options to improve crop productivity by improving crop yield potential. Crop yield potential can be improved via single process (green) optimisation, macro‐process (blue) optimisation or a whole plant improvement (e.g. by integrating the optimisation of multiple processes in parallel, such as photosynthesis and leaf longevity). N = nitrogen, C = carbon. The mapping of the options to improve crop yield potential was performed within the CropBooster‐P project (https://www.cropbooster‐p.eu/) (Baekelandt et al., 2022; Harbinson et al., 2021). Within the project, a similar mapping was done to identify the determinants of nutritional quality (Scharff et al., 2021) and sustainability (Gojon et al., 2022).

In this review, we present an overview of the key biological processes underlying crop yield potential that could contribute to the futureproofing of our current crops and that could be further exploited to improve crop productivity and safeguard future food security (Figure 1). More specifically, we describe a subset of plant traits and their genetic basis that contribute to yield potential, including photosynthesis, nutrient partitioning and remobilisation, leaf longevity, seed filling and plant organ growth and development. To go further, crop yield *potential* is defined here as yield in the absence of limitations by input, disease or suboptimal growing conditions. The conversion of radiation to dry matter (radiation use efficiency or RUE) and the partitioning of acquired resources can be closely related to yield potential in these conditions. Hence our selection of sub‐traits is based on this principle. Future perspectives are presented for each of these areas.

## 2 PHOTOSYNTHESIS: LIGHT CAPTURE AND THE EFFICIENCY OF CONVERTING LIGHT INTO PLANT BIOMASS

The primary determinant of crop biomass production is cumulative net photosynthesis over the growing season (Ort et al., 2015), where photosynthesis is defined as a plant process using the energy from light to convert carbon dioxide (CO_2_) and water (H_2_O) into oxygen (O_2_) and carbohydrates. The carbohydrates produced are used by the plant for growth and development. In addition, carbohydrates provide precursors for a range of diverse molecules including hormones, lipids and amino acids, and thus actually underpin all aspects of plant metabolism. Despite its importance, in agriculture photosynthesis has a ‘real world’ efficiency well below its theoretical maximum (Zhu et al., 2008), with only ~2% and 3% of the energy from sunlight being converted into biomass for current C_3_ and C_4_ crops, respectively, when grown under favourable conditions (Yin & Struik, 2015). Free‐air CO_2_ enrichment (FACE) experiments indicate that raising photosynthetic performance may increase crop yields (Ainsworth & Long, 2005; Long et al., 2006). Because photosynthesis is an energy‐transducing metabolic process in which CO_2_ fixation occurs via coordinated activities at various levels of biological organisation, including cells, organs, whole plants and communities (Long et al., 2015), numerous potential targets encompassing diverse metabolic and physical processes have been identified that could be exploited to increase photosynthesis and crop yield. A selection of the most promising are highlighted below.

### 2.1 Recovery from photoprotection and light induction of the Calvin cycle

Absorbed light energy can be in excess of what is required for photosynthesis. When a high proportion of chlorophyll molecules remain in an excited state and the electron transport system is highly reduced, then there is a greater risk of energy being transferred to oxygen, producing the more energetic and reactive oxygen species (ROS; Møller et al., 2007). These ROS can damage the photosynthetic machinery and membranes of the plant if they are not removed, and in particular when they are formed in the photosystem II (PSII) reaction centre, they can damage the reaction centre, resulting in a temporary (hours/days time range) loss of photosynthetic efficiency until the damaged reaction centre is repaired (Aro et al., 1993; Harbinson et al., 2022; Long et al., 1994; Murchie & Ruban, 2020). The overreduction of PSII can increase the likelihood of photoinactivation, that is the functional closure of reaction centres, leading to a decline in PSII activity. In addition, it is energetically costly to resynthesise PSII after damage by ROS (Li et al., 2018; Miyata et al., 2012). One way of protecting the plants from the negative consequences of excess radiation and the damaging effects of ROS is by means of enzymatic and non‐enzymatic antioxidants. In conditions where chlorophyll *a* excited state lifetimes increase due to the limitations imposed on PSII photochemistry by relatively slow electron transport, a further photoprotective mechanism engages to quench this excited energy. This protective mechanism limits the increase in chlorophyll *a* lifetime and reduces the pressure on blocked PSII reaction centres, as such limiting the formation of ROS in the first place. The activation of this process involves the protonation of the protein PsbS and the deepoxidation of the xanthophyll pigment violaxanthin to zeaxanthin, which drive rearrangements within the antenna systems of PSII that result in the dissipation of excitation energy as heat; a process known as non‐photochemical quenching (NPQ; Ahn et al., 2008; Havaux & Niyogi, 1999; Müller et al., 2001; Ruban, 2016). Xanthophylls are made via the methylerythritol phosphate (MEP) pathway that also produces volatile isoprenoids and hormones. These latter compounds may also have an important antioxidant effect specifically protecting the photosynthetic apparatus and often interacting with each other (Dani & Loreto, 2022). Emission of isoprene, the most abundant plant‐made volatile organic compound, may help to provide stable and homogeneous distribution of the light‐absorbing centres and more elastic thylakoid membranes (Pollastri et al., 2019, 2021). The NPQ process, while effective, has a drawback: it fully engages and disengages relatively slowly, which can result in limitations to assimilation in a fluctuating light environment, as occurring in the plant canopy.

As a determinant of yield, canopy photosynthesis is ultimately the product of leaf photosynthesis affected by spatial and temporal variation in light intensity and biochemical capacity. In a crop canopy, the light available for photosynthesis fluctuates continuously from a high (and even saturating) irradiance to light‐limiting irradiance, due to, for instance, clouds and self‐shading (Long et al., 2022). These changes can take place within the seconds time range (Durand et al., 2021) and can, in part, be relieved through changes in canopy structure that facilitate an improved canopy light environment (Araus et al., 2021; Burgess et al., 2015, 2017; Richards et al., 2019). Ideally, the photosynthetic processes would respond immediately to these rapid light fluctuations, but this is not the case. Following a transition from high to low light irradiance, the photoprotective NPQ engaged under high irradiance disengages relatively slowly, resulting in a momentary loss of light use efficiency for assimilation and therefore of potential carbon (C) gain by the plant (Burgess et al., 2015; Harbinson et al., 2022; Hubbart et al., 2012; Long et al., 1994; Murchie & Ruban, 2020; Werner et al., 2001; Zhu et al., 2004). Despite photoprotection being indispensable for plant survival, *in silico* simulations of photosynthesis in crop canopy‐like conditions have highlighted that a faster tuning of NPQ in response to changing light conditions may be important to improve crop production potential (Wang, Burgess, et al., 2020; Zhu et al., 2004). Bioengineering of an accelerated response to natural shading events in *Nicotiana tabacum*, achieved by overexpressing genes involved in the reversible conversion of zeaxanthin to violaxanthin (violaxanthin deepoxidase and zeaxanthin epoxidase), and the enzyme PsbS, resulted in increased leaf CO_2_ uptake and plant dry matter productivity and yield of 14‐25% in the field (Kromdijk et al., 2016; De Souza et al., 2022). Upregulating PsbS in rice leaves had a minimal effect on growth in constant light environment conditions (Hubbart et al., 2012). Under more realistic conditions, when considering a full canopy in fluctuating light, rice plants with increased PsbS and lower photoinhibition demonstrated higher radiation use efficiency and yield, indicating that fluctuating field conditions are crucial when determining productivity (Hubbart et al., 2018).

When subjected to an increase in irradiance, there is a delay in achieving a maximal photosynthetic rate, because this depends on an increase in metabolite pools and an activation of enzymes of the Calvin cycle, and on stomatal opening, all of which take time (Harbinson et al., 2022). The activation of ribulose‐1,5‐bisphosphate carboxylase/oxygenase (Rubisco), the CO_2_‐fixing enzyme, by Rubisco activase (Rca) is particularly slow (Hammond et al., 1998; Soleh et al., 2016; Woodrow & Mott, 1989) and there is strong evidence to improve photosynthetic efficiency under different circumstances, including fluctuating light, by targeting Rubisco (Harbinson et al., 2022). Modelling studies in wheat have indicated that the slow adjustment of photosynthetic biochemistry during shade‐to‐sun transitions reduces flag leaf photosynthesis by about 21% (Taylor & Long, 2017). Overexpression of *Rca* from maize in rice results in a slightly increased speed of photosynthetic induction at 25°C (Yamori et al., 2012) and overexpressing both *Rubisco* and *Rca* results in an increase in rice photosynthesis and yield at high temperatures (Qu et al., 2021). There is also evidence for significant genetic variation underlying the speed at which Rubisco carboxylation activity (*V*_cmax_) increases following a change from low‐ to high‐light conditions in wheat (Salter et al., 2019), and slow Rubisco deactivation speed may be linked to yield losses under fluctuating light conditions (Taylor et al., 2022). In line with this, there was considerable genotypic variation within the 37 parental lines of a nested association mapping in soybean, displaying variation in the speed of photosynthetic induction upon shade‐to‐sun transitions (Soleh et al., 2017). An *in silico* study showed that the CO_2_ assimilation loss attributable to photosynthetic induction ranged between 2 and 17% for these genotypes (Wang, Burgess, et al., 2020). Finally, substantial variation in rates of photosynthetic induction has been demonstrated in rice that may be limited by biochemistry (Acevedo‐Siaca et al., 2021).

While research on photosynthesis has largely focused on rates of CO_2_ uptake under steady‐state light conditions, it is increasingly apparent that improvements to photosynthesis require an understanding of how dynamic changes in the environment determine productivity. Future research efforts require a full characterisation of the light environment and the response by plants in field settings. It is still unclear how variation in light conditions, including the speed and magnitude of changes in intensity and spectral quality, quantitatively influence dynamic photosynthesis processes in the field, and how this might apply to diverse crop architectures (Burgess et al., 2021; Durand et al., 2021). Modelling approaches could provide one route towards resolving this, through advances in methods that represent 3‐dimensional plant structure and apply light simulations *in silico* (Burgess et al., 2017; Townsend et al., 2018). Furthermore, while the effects of environmental fluctuations tend to focus on short‐term fluctuations in the intensity of irradiance, changes in intensity will often be accompanied by changes in spectrum and not all wavelengths are equally good at driving photosynthesis (Hogewoning et al., 2012). Changes in irradiance will likely also be accompanied by other changes, such as leaf temperature, which will have their own effects on the photosynthesic processes. Environmental changes can also be long‐term and not just the short‐term (minutes to hours) fluctuations that are the focus of much current research. Longer term environmental changes are likely to be accompanied by longer term changes in gene expression and remodelling of photosynthetic and other metabolic pathways or processes (Chow et al., 1990; van Rooijen et al., 2018), though currently also largely underexplored.

### 2.2 Antenna pigment composition and electron transport rates

The light‐harvesting antennae contain the chlorophyll molecules that capture light energy to feed into the photosystem centres (PSI and PSII), thereby driving electron transport. At the top of the canopy, more light energy is often absorbed than can be used in the photosynthesis process (Walker et al., 2018). This may be beneficial in the wild, where a plant capturing more light by its upper leaves blocks light transmission to competing understory plants. In a mixed crop culture, however, more equal distribution of the light‐harvesting capacity of leaves across the entire canopy may increase productivity (Friedland et al., 2019; Walker et al., 2018; Wu et al., 2020). Several modelling studies have shown that improving photosynthetic electron transport components is crucial for increasing canopy photosynthesis (e.g. Yin et al., 2022). More specifically, modelling approaches suggest that when reducing the antenna size of PSII or the total leaf chlorophyll in a more balanced way (i.e. affect PSI and PSII to the same extent) and as such reducing light absorption, upper canopy leaves could not only save on resources but also allow more light to reach lower canopy leaves and therefore improve photosynthesis at canopy level (Ort et al., 2011), as shown in rice (Gu et al., 2017), wheat (Hamblin et al., 2014) and soybean (Walker et al., 2018). The high chlorophyll content in contemporary crops may be linked to the breeder's selection for high leaf nitrogen (N). Chlorophyll‐*a*‐oxidase has, for instance, been reported to be related to antenna size (Friedland et al., 2019; Masuda et al., 2003; Slattery et al., 2017) and could thus be a target to improve canopy light distribution and therefore canopy light use efficiency and assimilation. The photosynthetic apparatus, in particular Rubisco, constitutes the major pool of N in leaves and thus high N content is essential for photosynthesis and plant growth (Warren et al., 2000; Zhu et al., 2008). Accordingly, dark green leaves, which act as an indicator of N content, were frequently selected for in the past, based on expectations this would increase yields due to improved C fixation (Friedman et al., 2016). It appears that this selection may have led to crops with a suboptimal light distribution (Walker et al., 2018).

The cytochrome *b*_*6*_ *f* (cyt *b*_*6*_ *f*) complex connects electron transport from PSII to PSI and is under non‐light‐limited conditions the rate‐limiting step in the electron transport chain. When photosynthesis is not light‐ or rubisco‐limited or limited by the regeneration phase of the Calvin cycle, electron transport (and thus the cyt *b*_*6*_ *f* complex) will limit photosynthesis and be the cause of the loss of photosynthetic light use efficiency. Increasing the activity of the cyt *b*_*6*_ *f* complex may therefore also increase the photosynthetic rate (von Caemmerer & Furbank, 2016). In line with this, it has been shown that overexpressing the Rieske FeS protein (PetC) component of the cyt *b*_*6*_ *f* complex in Arabidopsis results in a concomitant increase in the levels of the cyt f (PetA) and cyt *b*_*6*_ (PetB) core proteins of the cyt *b*_*6*_ *f* complex (Simkin et al., 2017; Yamori et al., 2016). This leads to an increase in the levels of proteins in both PSII and PSI and has a significant impact on the quantum efficiency of both photosystems, the electron transport, biomass and seed yield (Simkin et al., 2017). Similar results have been seen in the C_4_ bioenergy grass, *Setaria viridis* (Ermakova et al., 2019). Altogether, these examples demonstrate the potential of fine‐tuning the electron transport processes to increase crop productivity.

There are new opportunities to enhance light harvesting processes and electron transport rates to improve photosynthesis at both cellular and canopy scales. Although canopy light interception is generally not considered a major limitation to crop yield, the distribution of photosynthetic activity can be influenced by enhanced light penetration as a result of leaf angle or movement (Burgess et al., 2016, 2021). In the future, approaches to optimise light use efficiency by electron transport processes should be combined with alterations in canopy architecture to further enhance the distribution of light transmission. We must also underline that increasing the photosynthesis rate of crops without improving nutrient uptake and use efficiency at the same time is unlikely to have a positive impact on yield (Sinclair et al., 2019; Yin et al., 2022). Because nutrients like N and phosphorus (P) are essential components of key cell compounds and, particularly for N, among the main drivers of leaf growth and the interception of solar energy, dramatic increases in crop biomass could only be achievable if such nutrients, and solvent water, are sufficient.

### 2.3 The photorespiratory cost, C_4_ crops and other CO_2_ ‐concentrating mechanisms

Rubisco is responsible for the fixation of atmospheric CO_2_ and is the first step in producing organic carbon compounds. As such, Rubisco plays a central role in determining the rate of CO_2_ fixation, although some of its characteristics may severely compromise photosynthetic productivity. Rubisco can react with both CO_2_ and O_2_ as substrates. Despite catalytic properties favouring CO_2_ as a substrate, 20 to 50% of Rubisco reactions occur with O_2_ in a process called photorespiration, leading to both a waste of energy and a loss of fixed C (von Caemmerer, 2020). The initial product of the oxygenation reaction, 2‐phosphoglycolic acid (2‐PG) inhibits, amongst others, some enzymes of the Calvin‐Benson cycle and hence must be rapidly removed and recycled. Photorespiration is considered one of the most energy‐demanding metabolic processes in plants (Sonnewald, 2018) and regional scale models have shown that photorespiration decreases current US soybean and wheat yields by 36% and 20%, respectively (Walker et al., 2016). Climate change is expected to further influence the balance between photosynthesis and photorespiration. On one hand, CO_2_ concentration is increasing, favouring carboxylation and the Calvin‐Benson cycle. On the other hand, increasing temperature is decreasing the relative CO_2_/O_2_ solubility, making O_2_ more available to Rubisco (Walker et al., 2016). Accordingly, photorespiration rates and their negative impact on crop yield are projected to increase in the coming decades due to global warming (Dusenge et al., 2019).

Since its discovery, reducing photorespiration has been seen as an important target for crop improvement (Zelitch & Day, 1973). One strategy is to decrease the costs associated with photorespiration by introducing novel metabolic pathways that efficiently recycle the products of Rubisco oxygenation (Peterhansel et al., 2013). In Arabidopsis, introducing photorespiratory bypasses such as bacterial glycolate‐metabolising enzymes or a glycolate decarboxylation pathway that either recycle 2‐PG to 3‐phosphoglyceric acid or fully decarboxylate it within the chloroplast significantly increases photosynthetic rates and biomass production in growth room and greenhouse experiments (Kebeish et al., 2007; Maier et al., 2012). *In silico* simulations of these alternative pathways demonstrated that to maximise the benefits for crops grown under field conditions, optimisation should target both maximum flux through the alternative pathway as well as minimal flux through the current photorespiratory pathway (Xin et al., 2015). This approach has been pursued in tobacco, where three distinct alternative pathways were evaluated, recently reviewed in Eisenhut et al. (2019). All three pathways start with glycolate, which is formed from 2‐PG by the plant native enzyme 2‐PG phosphatase. They differ in downstream biochemical processes and the number of transgenes required: two, three or five. Each of the alternative pathways has been combined with the repression of the plastidal glycolate/glycerate transporter PLGG1 to reduce the efflux of glycolate from the chloroplast and increase the efficiencies of the synthetic bypasses. Field trials with engineered tobacco plants identified the two‐transgene alternative pathway in combination with the repression of PLGG1 as the most effective strategy, and the plants displayed significant biomass increases compared with controls (South et al., 2019). Promising biomass increases in 14 to 35% and grain yield increases in 7%–27% were also reported following the introduction of an alternative photorespiratory pathway in rice, which suppressed photorespiratory rates by 18%–31% compared with the controls (Shen et al., 2019; Wang, Shen, et al., 2020). Similar promising results were reported for the oilseed crop *Camelina sativa* (Dalal et al., 2015), suggesting that this approach could be exploited to improve yield in a wide range of C_3_ crops (South et al., 2018). It is however unclear whether plants with alternative pathways will maintain performance advantages, or even experience adverse effects relative to controls, when grown under suboptimal conditions.

As an alternative strategy to reduce photorespiration, the Rubisco oxygenation reactions could be decreased by increasing the CO_2_ concentration around the enzyme. In C_3_ crops, CO_2_ concentration is expected to be much lower at the Rubisco active sites than in the atmosphere, because of stomatal and mesophyll resistances to CO_2_ diffusion towards the chloroplasts. Where these resistances are greater, photosynthesis will be diminished and photorespiration increased (Iñiguez et al., 2020). The chloroplast CO_2_ concentration is generally further reduced when plants are exposed to abiotic stresses linked with stomatal closure (Flexas et al., 2004, 2006). To increase CO_2_ concentration at Rubisco sites, some photosynthetic algae, bacteria and plants have evolved C‐concentrating mechanisms (CCMs), such as C_4_ photosynthesis (Sage, 2004). In C_4_ photosynthesis, a two‐step process of CO_2_ assimilation is spatially distributed within cells or between cells within leaf tissues. In current C_4_ crop plants like maize, sorghum, sugarcane and millet, gaseous CO_2_ is initially fixed by phosphoenolpyruvate carboxylase (PEPC) into C_4_ acids, which are then transported to deeper, gas‐tight, bundle sheath cells where decarboxylation occurs, releasing CO_2_ for subsequent recapture by Rubisco (Sage, 2004). In bundle sheath cells of C_4_ crops, there is also no or only little O_2_ production (Westhoff et al., 1990), further increasing the relative CO_2_ concentration near Rubisco (Sage et al., 2012). Because there is an approximately tenfold increase in the CO_2_ concentration within the bundle sheath cells, Rubisco oxygenation reactions are almost entirely suppressed (Carmo‐Silva et al., 2008).

Converting C_3_ into C_4_ crops is an ambitious goal requiring both anatomical and biochemical changes and with components of bundle sheath and mesophyll tissues expressed and regulated correctly to be functional (Ermakova et al., 2020; Lin et al., 2020). Many of the necessary 'building blocks' are already available within C_3_ crops and recent developments in bioinformatics and biotechnology make success more realistic (https://c4rice.com/). Alternative approaches that do not require anatomical changes are to add cyanobacterial, algal or anthocerote CCMs to crop chloroplasts. Unlike the CCM of C_4_ plants, which relies on a biochemical pump, these CCMs work by means of biophysical CO_2_ or bicarbonate pumps. In the cyanobacterial CCM, Rubisco is packed within a protein‐bound structure called the carboxysome, while in the algal or anthocerote CCM, Rubisco aggregates to form a structure called the pyrenoid (Atkinson et al., 2020; Long et al., 2015; Price et al., 2013). Creating a high CO_2_ concentration in the carboxysome or pyrenoid would reduce the energetic loss to photosynthesis due to the oxygenation of ribulose‐1,5‐bisphosphate and allow Rubisco to work more efficiently by producing a CO_2_ concentration closer to saturation for that enzyme. Modelling studies suggest that this could increase crop yield by approximately 30% (McGrath & Long, 2014), or by even higher percentages if the energy requirement of cyanobacterial CCMs is confirmed to be lower than the ATP‐costly C_4_ crop CCMs (Yin & Struik, 2017). Substantial progress has already been made by introducing functional cyanobacterial Rubisco into crops and by expressing both alpha and beta forms of the carboxysomes in plants (Lin, Occhialini, Andralojc, Devonshire, et al., 2014; Lin, Occhialini, Andralojc, Parry, & Hanson, 2014; Long et al., 2018; Wang, Yan, et al., 2019). However, to be effective, the rest of the cyanobacterial system must also be present and functional (Atkinson et al., 2020). The research field of CCMs is relatively new and may offer great opportunities to improve the photosynthetic rates and thus plant yield.

While not a CCM, increases in the diffusion of CO_2_ from the free air surrounding the leaf towards the site of carboxylation would increase the CO_2_ concentration at the site of carboxylation and thus increase the photosynthetic rates. This diffusive pathway includes the boundary layer, stomatal and mesophyll conductance. Increases in any of these would, all other things being equal, increase the CO_2_ concentration in the chloroplast. Mesophyll conductance is not only a major diffusional limitation for CO_2_ (Warren, 2008), but improving mesophyll conductance would also allow an increase in the photosynthetic water use efficiency for C_3_ plants (Flexas et al., 2012, 2013). Physical (e.g. cell wall and membrane and chloroplast surface area and movement) and biochemical (e.g. aquaporins and carbonic anhydrase availability) factors may both contribute to limit CO_2_ concentration in the chloroplasts, hence limiting photosynthesis (Evans, 2021). Increasing mesophyll conductance has been proposed as a target for improving photosynthesis and crop yields (Ren et al., 2019). Little, however, is known about the underlying genetics of mesophyll conductance (Flexas et al., 2012, 2013; Ren et al., 2019). An increased density of leaf venation has also been associated with a higher rate of photosynthesis (Boyce et al., 2009; Brodribb et al., 2007).

## 3 NUTRIENT PARTITIONING AND REMOBILISATION, LEAF LONGEVITY AND SEED FILLING

An important component of plant productivity is the partitioning of organic C and N among the various plant organs (Evans & Poorter, 2001; Yadav et al., 2015). Nutrient partitioning requires export from the sites of primary uptake and assimilation, transport throughout the plant by phloem and xylem, and import into the various sink organs such as seeds, taproots and rhizomes (Tegeder & Masclaux‐Daubresse, 2018). In perennial trees and grasses, for instance, stems and roots serve as reservoirs storing C and N. The major energy and C storage compounds of plants are starch, fructans and oils, whereas the N storage compounds are mainly proteins. Proteins represent 10%–40% of the total seed weight depending on the plant species (Baud et al., 2008). The reserves that accumulate after satisfying the demands of plant growth and metabolism determine the quality of harvested plant products for human and animal food (Pask et al., 2012). Increasing the capacity of plants to store nutrients in non‐photosynthetic organs, like stems or tubers, may extend the duration of photosynthesis and be one way to increase nutrient use efficiency (Martre et al., 2007).

For most species, seeds are also main storage organs. They typically accumulate large reserves of nutrients that will significantly support germination and the early stages of plant development in all but a few exceptional cases. Seed filling therefore is highly important for plant fitness and is essential for food security, because it determines both seed size and nutritional quality. In crops, efficient seed filling is a key factor controlling yield (El‐Zeadani et al., 2014; Houshmand et al., 2022; Reynolds et al., 2021; Sehgal et al., 2018). Several crucial steps in N allocation need to be taken into consideration to improve crop productivity and nutritional quality of harvested products (Paul et al., 2017). These include optimising source‐sink ratios, promoting efficient translocation of assimilates to harvestable organs and optimising the balance between biosynthetic activities in vegetative organs and nutrient remobilisation from senescing organs towards reproductive organs (Havé et al., 2017). Here, we present some of these processes and the underlying molecular players that could be exploited to improve intrinsic crop yield.

### 3.1 Carbohydrate allocation to harvestable parts

Crop productivity can be improved by targeting C allocation towards the harvestable plant organs, such as stems, tubers, roots, reproductive organs and seeds, by directly modifying genes controlling the processes of carbohydrate accumulation in source and sink organs (Foulkes et al., 2022; Lu et al., 2020; Murchie et al., 2022; Oszvald et al., 2018). Trehalose 6‐phosphate (T6P) is the phosphorylated precursor of the non‐reducing glucose disaccharide trehalose. It is known that T6P acts as a signal of sucrose availability that regulates plant growth and development (Fichtner & Lunn, 2021; Paul et al., 2018). T6P has been shown to increase photosynthetic rates in maize, *N. tabacum* and rice (Li et al., 2022; Oszvald et al., 2018; Pellny et al., 2004). Low levels of T6P are thought to act as a starvation signal that stimulates sucrose flux towards the sinks (Oszvald et al., 2018). Altering the levels of T6P in wheat, using genetic variations in trehalose phosphate synthase (*TPS*) and trehalose phosphate phosphatase (*TPP*) genes, was identified as a promising strategy to enhance sink strength and source‐sink interactions (Lawlor & Paul, 2014; Lyra et al., 2021). Overexpression of *TPP*, encoding a T6P phosphatase, in the phloem vasculature of female reproductive tissues of maize, decreases T6P levels in developing cobs and results in a relocation in sucrose and amino acids from cob pith towards developing kernels (Oszvald et al., 2018). Moreover, targeting the T6P regulation results in increased maize yield (Nuccio et al., 2015). A *TPP* gene in wheat was found to underlie a quantitative trait locus (QTL) associated with grain size (Zhang et al., 2017) and applying a chemically modified plant‐permeable analogue of T6P to wheat ten days after anthesis increases both grain size (up to 20%) and starch accumulation (Griffiths et al., 2016).

T6P inhibits the feast‐famine protein kinase Sucrose non‐fermenting 1 (Snf1)‐RELATED KINASE 1 (SnRK1), which is a master gene of sucrose sensing. SnRK1 is activated upon C starvation or stress. Its antagonist, the TARGET OF RAPAMYCIN (TOR) kinase is activated upon nutrient supply (Dobrenel et al., 2016). In this way, SnRK1 and TOR play paramount roles in the regulation of plant growth in response to the nutrient status of plant tissues (Burkart & Brandizzi, 2021; Ingargiola et al., 2020; Li et al., 2021). Interestingly, the SnRK1/TOR complex not only controls starch accumulation but also lipid synthesis and nutrient recycling through autophagy (Baena‐González & Hanson, 2017). SnRK1 interacts with the ATAF1 transcription factor, which integrates C starvation responses. ATAF1 induces the expression of autophagy genes that control nutrient recycling but is also a repressor of the GOLDEN2‐LIKE1 (GLK1) transcription factor, which is involved in chloroplast maintenance. It is thus likely that ATAF1 is involved in the fine‐tuning of the shift from C and N primary assimilation to nutrient recycling (Garapati, Feil, et al., 2015; Garapati, Xue, et al., 2015; Kleinow et al., 2009). As such, manipulating the TOR/SnRK1 balance or activities would be a way to control nutrient assimilation and storage on one hand, as well as nutrient recycling and mobilisation on the other hand (Liu & Bassham, 2010).

The altered allocation of resources upon modulation of the T6P/SnRK1 pathway can be explained by the upregulation of SWEET sucrose transporters (Oszvald et al., 2018). *SWEET4* genes encode hexose transporters involved in the uptake of hexoses produced by cell wall invertases in developing seeds (Sosso et al., 2015). These genes have been targets for selection during domestication, and modulation of their expression and/or activity is an alternative strategy to increase carbohydrate uptake into developing seeds (Sosso et al., 2015). Besides SWEET proteins, the sucrose transporters SUT and SUC are involved in apoplastic loading (Bürkle et al., 1998). In the *Atsuc2‐4* mutant, phloem loading can be rescued upon expression of *AtSUC1*, *AtSUC2* or *ZmSUT1* (Dasgupta et al., 2014). In addition, apoplastic unloading needs to be enhanced, for instance in seeds. Overexpression of *AtSTP13*, encoding a sugar transporter, increases glucose uptake by Arabidopsis seeds, resulting in an increase in plant biomass (Schofield et al., 2009). Conversely, RNAi‐mediated knock‐down of the high‐affinity hexose transporter gene *LeHT* leads to a massive decrease in fruit hexose accumulation in tomato (McCurdy et al., 2010). The sucrose phloem loading mechanism appears to be conserved across many crops (Braun et al., 2014) and understanding the underlying mechanisms may thus offer great potential to improve yield of various crops. Since most crops still seem to have sink limitation during seed filling, breeders will need to keep improving C allocation to harvestable parts. Additionally, it is recognised that C assimilate availability through remobilisation of C storage should prolong starch synthesis and increase C allocation to seeds by extending the duration of seed growth. For example in wheat, breakdown of fructans feeds growing seeds: fructan exohydrolase 1‐FEH v3 mapping on chromosome 6B is a useful marker for fructose breakdown (Khoshro et al., 2014; Zhang et al., 2008).

Optimising the source‐sink transfer is a promising and feasible way to optimise photosynthesis and improve the productivity of crops (Dingkuhn et al., 2020; Oszvald et al., 2018). In many crop species, photosynthesis during the seed‐filling period appears to be responsive to increases in seed sink strength through genetic effects that increase seed number, even in modern cultivars with already high seed numbers (e.g. Acreche & Slafer, 2009). More specifically, the T6P/SnRK1/TOR pathway might be amenable for yield improvement (Baena‐González & Hanson, 2017; Paul, 2021) and several T6P pathway genes are amongst those associated with domestication improvement in maize (Hufford et al., 2012). Interventions that modify T6P through genetic modification in maize (Nuccio et al., 2015), chemical application in wheat (Griffiths et al., 2016) and natural variation in wheat and rice have shown that the T6P pathway is not yet optimised in crops and thus has potential for further yield improvement (Lyra et al., 2021; Paul et al., 2020). To establish which changes can be made to further improve crop yield and resilience, it will remain interesting and important to understand how the T6P pathway, and likely also other pathways involved in source‐sink transfer, can be modified through breeding.

### 3.2 Regulation of senescence and nitrogen remobilisation

During seed formation, C dedicated to seed filling is mainly provided by photosynthetic C fixation occurring in leaves and in the fruit tissues, such as pod walls in legumes, silique envelopes in Brassicaceae, and glumes and awns in cereals (Araus & Tapia, 1987; Cliquet et al., 1990; Tambussi et al., 2021). The lifespan of the leaf controls the duration of photosynthetic C fixation and primary N assimilation, establishing the total C and N uptake by the crop, strongly impacting seed yield. The timing and rate of the leaf senescence then determine nutrient recycling and mobilisation, both important for seed filling with N and other nutrients (Masclaux‐Daubresse et al., 2010). Thus, the process of seed filling and the accumulation of major seed reserves are intimately linked with the senescence of the source tissues in many plant species (Havé et al., 2017; Woo et al., 2019). Leaf senescence is also controlled by endogenous factors including phytohormones and metabolic status, and exogenous factors such as shading, drought or nutrient deficiencies (Jordan et al., 2012; Kim et al., 2018). Cytokinin hormones are endogenous inhibitors of leaf senescence (Gan & Amasino, 1995). Various attempts have been made to delay senescence by altering cytokinin levels as a way to increase biomass and seed yield (Dani et al., 2022). Interestingly, cytokinins regulating leaf and plant senescence seem to be intimately connected to isoprenoid metabolism (Dani et al., 2022) and this may be one reason why only deciduous leaves emit isoprene (Loreto & Fineschi, 2015). In various model and crop species, overexpression of the *IPT* cytokinin synthesis gene in senescing tissues has been obtained using promoters of senescence‐associated genes (Guo & Gan, 2014; Jordi et al., 2000). For instance, *SENESCENCE‐ASSOCIATED GENE 12* (*SAG12*) from Arabidopsis, *SENESCENCE‐ENHANCED 1* (*SEE1*) from maize, *SENESCENCE‐ASSOCIATED RECEPTOR‐LIKE KINASE* (*SARK*) from bean, CYSTEINE PROTEASE (*Ghcysp*) from cotton and *SENESCENCE‐ASSOCIATED GENE 39* (*SAG39*) from rice have been used to delay leaf senescence and increase plant performances (Guo & Gan, 2014). In a different approach, a delay in senescence has been obtained by lowering the senescence‐promoting hormones such as salicylic acid (SA) by expressing the bacterial SA hydroxylase *NAPHTHALENE CATABOLIC GENE* (*NahG*) or by mutating the isochorismate synthase gene *SALICYLIC ACID INDUCTION DEFICIENT 2* (*SID2*; Abreu & Munné‐Bosch, 2009). The linked reduction in SA levels leads to a marked increase in biomass and seed production, indicating that alterations in SA levels could be exploited to increase crop yield (Abreu & Munné‐Bosch, 2009).

At the transcriptional level, leaf senescence is governed by several transcription factors mainly belonging to the NAC and WRKY protein families (Borrill et al., 2019; Cormier et al., 2015, 2016; Derkx et al., 2021; Distelfeld et al., 2012; Lee & Masclaux‐Daubresse, 2021). Amongst these proteins is ATAF1 discussed in relation to carbohydrate allocation to harvestable parts and a NAM‐B1 transcription factor that has been identified from a quantitative genetic study in durum wheat. The *NAM‐B1* gene, also known as the *Gpc‐B1* locus on chromosome 6B of bread wheat, is a master gene controlling leaf senescence, grain yield and protein content (Uauy et al., 2006; Waters et al., 2009). Whereas modern wheat varieties rarely carry a functional *NAM‐B1* allele, the ancestral wild wheat allele of *NAM‐B1* (*Triticum turgidum* ssp. *Dicoccoides DIC* allele) accelerates senescence and increases nutrient (N, Fe and Zn) remobilisation from leaves to developing grains (Distelfeld et al., 2014). An analysis of published data revealed that the presence of a functional copy of the *NAM‐B1* gene is associated with increased protein and micronutrient content in grains, though with a marginally negative effect on yield (Pearce et al., 2014; Tabbita et al., 2017). Effects of *NAM‐B1* alleles were also found in barley, and better performing alleles are used in several cereal breeding programmes (Parrott et al., 2012). Some other genes of the same family, like the homologous NAM‐A1 (with its functional allele NAM‐A1a), could be used to improve wheat grain protein content while maintaining yield (Alhabbar et al., 2018; Cormier et al., 2015; Derkx et al., 2012). Several other leaf senescence regulatory genes, identified in Arabidopsis and rice, were shown to confer functional stay‐green phenotypes and yield improvements, for example the *Ghd7* (Grain number, plant height and heading date 7) and *ONAC2* genes of rice (Lee & Masclaux‐Daubresse, 2021; Mao et al., 2017; Singh et al., 2021; Wang et al., 2015).

Seed filling with N can be achieved through post‐flowering N uptake from the soil during seed formation and through remobilisation of organic N from senescing vegetative tissues. Because seed storage protein content largely relies on N remobilisation (Masclaux et al., 2001), the onset of leaf senescence and its rate are major factors for grain protein content (Thomas et al., 2002; Thomas & Howarth, 2000; Van Oosterom, Borrell, et al., 2010; Van Oosterom, Chapman, et al., 2010). The photosynthetic apparatus is known to be the largest protein reserve and N source in leaves for remobilisation (Peoples & Dalling, 1988; Warren et al., 2000; Zhu et al., 2008). Thus, a trade‐off between photosynthesis and senescence leads to a trade‐off between maximising C assimilation versus N remobilisation for seed production and seed filling (Yin et al., 2022). As a consequence, frequently selected‐for‐stay‐green phenotypes are not always associated with higher yields because maintenance of the photosynthetic apparatus is counterproductive for N remobilisation towards developing seeds (Thomas & Ougham, 2014).

Studies of the metabolic pathways and cellular mechanisms controlling nutrient fluxes from senescing leaves towards the seeds have mainly focused on N‐metabolism enzymes, ATG proteins involved in macro‐autophagy machinery and proteases (Havé et al., 2017; Lee & Masclaux‐Daubresse, 2021). Amongst them is the prominent role of macro‐autophagy in N remobilisation from leaves to the seeds, which has been demonstrated in several plant species as Arabidopsis, maize and rice (Guiboileau et al., 2012; Li et al., 2015). The macro‐autophagy machinery is a vesicular mechanism essential for the trafficking of cytoplasmic components to the lytic vacuole, where proteolytic activities will degrade them to release nutrients (Masclaux‐Daubresse et al., 2017). The induction of macro‐autophagy in senescing leaves has a fundamental role in (i) maintaining cell longevity by removing oxidised components that are potentially toxic and (ii) nutrient recycling by driving unwanted proteins and macromolecule to degradation in the vacuole, thus providing amino acids and sugars for remobilisation towards the seeds (Guiboileau et al., 2012; James et al., 2018, 2019; Li et al., 2015; Pružinská et al., 2017). Fine‐tuning of autophagy activity in leaves is essential to maintain leaf longevity. Increasing autophagy improves nitrogen use efficiency (NUE) in Arabidopsis and rice, because it facilitates the release of N metabolites in source tissues (Chen et al., 2019; Guiboileau et al., 2012; Yu et al., 2019). The nature of the transporters involved in the release of the products of autophagy from the vacuole and further from leaf cells, for phloem loading and export from senescing leaves to seed loading, has been poorly investigated so far. The LEUCINE‐HISTIDINE TYPE TRANSPORTER 1 (LHT1), which improves amino acid uptake at the root level, could also play a role in N remobilisation because it is also induced with senescence in leaves (Guo et al., 2020; Hirner et al., 2006; Wang, Yang, et al., 2019). The AAP8 AMINO ACID PERMEASE (AAP), which is involved in phloem loading of amino acids in source leaves, has been shown to control seed loading (Santiago & Tegeder, 2016; Zhang et al., 2010, 2015). The UmamiT transporters (UmamiT11, UmamiT28, UmamiT29 and UmamiT18) have been shown to control free amino acids levels in fruits and during seed development (Ladwig et al., 2012; Müller et al., 2015). Remobilisation of inorganic N during senescence might also be interesting for seed filling in plants that are able to store nitrate or ammonium in vacuoles. The NRT1.7 and NRT2.5 nitrate transporters and the Dur3 urea transporter are induced during senescence and remobilise nitrate and urea from Arabidopsis leaves to sink tissues during senescence (Bohner et al., 2015; Fan et al., 2009; Kojima et al., 2007; Lezhneva et al., 2014; Wu et al., 2014). Several transporters were identified as targets to improve N flux towards seeds, and manipulation of several nitrate and amino acid transporters successfully improved yield and NUE in several plant species (Tegeder & Masclaux‐Daubresse, 2018). Although the precise role of many transporters in phloem loading, unloading and xylem to phloem translocation is not well known, the concurrent activations of some of these transporters is a strategy to improve N flux towards seeds that deserves further research.

It is well known that amino acid catabolism occurs in senescing leaf tissues to support mitochondrial respiration through conversion to keto‐acids (Chrobok et al., 2016). The cytosolic GLUTAMINE SYNTHETASES (GS1) and ASPARAGINE SYNTHETASES (ASN) that are induced during leaf senescence are essential to reassimilate ammonium released from amino acid catabolism. These enzymes contribute to the synthesis of glutamine and asparagine that are the preferred amino acids for phloem loading (Havé et al., 2017; Lee, 2021; Moison et al., 2018; Xu et al., 2012). Manipulation of these enzymes is complex as they exist as multigenic families. Several studies performed in maize and rice report the positive effects of activation of these enzymes on plant productivity, which encourages their manipulation (Lee, Marmagne, et al., 2020; Lee, Park, et al., 2020; Martin et al., 2006).

The important limiting steps in N management for biomass and yield improvement are the capacities of plants to provide enough N at the right place and at the right time of development to support optimal growth of the plant organs. For that, the capacity of a plant to use structural proteins, enzymes and other macromolecules as N reservoirs in vegetative tissues is essential. Plants that have the capacity to efficiently degrade, recycle and translocate organic N from macromolecules without affecting cell longevity, need less inorganic N input. Such an ability requires the simultaneous modulation of the metabolic and physiological processes mentioned throughout this review, such as photosynthesis, senescence and nutrient transport and partitioning. In addition, because the photosynthetic machinery represents the main N reservoir in vegetative green tissues of most plants, leaf senescence has opposite effects on C fixation and N remobilisation.

The impact of the regulatory genes of the leaf senescence programme on photosynthesis, nutrient partitioning, nutrient translocation and grain production needs further investigation to understand the interaction of all these gene products controlling leaf longevity, chloroplast maintenance, plant growth and nutrient recycling throughout the plant's lifespan. Breaking this negative relationship to obtain plants that can maintain both C fixation and N recycling and mobilisation as long as possible is an interesting future research question for breeding strategies.

### 3.3 Oil/lipid metabolism

Many plant species, including model species such as Arabidopsis and crops such as sunflower and rapeseed, accumulate fatty acids as the principal energy source in seeds. Fatty acid production relies on sucrose produced through photosynthesis and transported to the seeds (Miray et al., 2021; Troncoso‐Ponce et al., 2016). Sucrose is hydrolysed to glucose and fructose, which are then converted to acetyl‐coenzyme A (CoA) via glycolysis. Acetyl‐CoA is then utilised for fatty acid biosynthesis in seed plastids, from which triacylglycerols (TAGs) are synthesised in the endoplasmic reticulum and accumulate in oil bodies (oleosomes). Manipulation of enzymes and transcription factors involved in TAG metabolism has been thoroughly explored, and several have increased oil concentrations in seeds (Kong et al., 2020; Troncoso‐Ponce et al., 2016; van Erp et al., 2014; Weselake et al., 2009). Other strategies for increasing oil content in seeds include manipulating chloroplast fatty acid transporters to increase seed oil accumulation (Li et al., 2020; Tian et al., 2018).

The pull, push and protect approach (Vanhercke, Petrie, et al., 2014) consists of the induction (push), the synthesis (pull) and the protection (protect) of TAG‐containing bodies (oil bodies) in plants. This approach was used to promote oil production and accumulation in vegetative tissues and especially in leaves. The concurrent overexpression in *N. tabacum* of (i) the Arabidopsis *WRINKLED1* (*WRI1*) gene that encodes a transcription factor that enhances the expression of genes involved in lipid synthesis, (ii) the *ACYL‐COA:DIACYLGLYCEROL ACYLTRANSFERASE1* (*DGAT1*) gene that promotes the formation of oil bodies, and (iii) the *OLEOSIN* gene that codes for a coat protein that defines and protects oil bodies results in the production of the ‘high oil’ tobacco lines that contained 15% more TAGs (dry weight) in their leaves (Marchive et al., 2014; Vanhercke et al., 2013; Vanhercke, El Tahchy, et al., 2014). Furthermore, Vanhercke et al. (2017) silenced the *SUGAR‐DEPENDENT1* (*SDP1*) gene encoding a lipase that degrades oil bodies to interrupt the first step of TAG turnover and overexpressed the *Arabidopsis thaliana* transcription factor LEAFY COTYLEDON 2 (LEC2) in the ‘high oil’ tobacco previously engineered. The *LEC2* master regulator of seed maturation and oil accumulation in seeds was expressed under the control of the senescence‐associated promoter *SAG12*, to minimise negative pleiotropic effects of constitutive *LEC2* expression (Kim et al., 2015). These new constructs increased TAG accumulation levels in the leaf tissues by 30%–33% relative to the wild type (Vanhercke et al., 2017, 2019). Several studies also demonstrate the positive effect of intercepted light and leaf senescence retardation and on seed oil content (Aguirrezábal et al., 2003; Andrianasolo et al., 2016). In addition to the efforts made to increase oil yield, studies aimed at improving oil quality (Napier & Graham, 2010). For instance, numerous biotechnological solutions were proposed to change oil composition to fit to the diversity of consumer demands (Haslam et al., 2016).

To summarise, successes have been achieved leading to increases in oil accumulation not only in seeds but also in vegetative tissues, offer new perspectives in producing high‐energy plant products. Plant metabolism is exceedingly plastic and capable of offering solutions that meet human needs in terms of oil quality and quantity, including nutrition, food processing, industrial processes and biofuel production. So far, however, this potential is underexploited because of limited understanding. To meet the future crop productivity demands, it is crucial to unravel the mechanisms and genetic regulation underlying oil and lipid metabolism, as well as their interconnections with other plant processes.

## 4 PLANT ORGAN GROWTH AND DEVELOPMENT

A key determinant of crop yield is organ growth and development, of which several aspects and their link with the photosynthetic and nutrient remobilisation processes were described earlier. Plant growth is controlled by complex, highly interconnected networks of regulators that integrate many different internal and external signals, including light, sugars, water availability and minerals (Hilty et al., 2021). These inputs are translated into distinct processes, such as the spatial organisation of plant tissues, the cell cycle and/or cell expansion, cell–cell communication and cell death.

### 4.1 Leaf growth and development

Leaves are often taken as model organs to elucidate various processes underlying organ growth and the underlying molecular pathways. In addition, leaves are the direct and main source of plant‐derived products and the predominant sites of photosynthesis. In their role as major C‐ and energy‐producing factories, leaves allow plants to sustain their growth, to complete their life cycle and to form other organs of agricultural importance, such as seeds and fruits (Barber, 2009; Demura & Ye, 2010; Tsukaya, 2013; Zhu et al., 2010).

Leaf development is a multifactorial and dynamic process, and distinct aspects of leaf development and the underlying molecular networks have been identified and reviewed extensively (Gonzalez et al., 2012; Hepworth & Lenhard, 2014; Nelissen et al., 2016; Nelissen & Gonzalez, 2020; Powell & Lenhard, 2012; Vercruysse et al., 2020). At a cellular level, the main mechanisms that contribute to leaf size and/or shape determination are (i) the number of cells recruited to the organ primordium (Efroni et al., 2010; Kalve et al., 2014; Reinhardt et al., 2000), (ii) the rate and (iii) duration of cell division (Andriankaja et al., 2012; Donnelly et al., 1999; Gonzalez et al., 2012; Vercruysse et al., 2020), (iv) the rate and (v) duration of cell expansion and (vi) the extent of meristemoid division, the re‐iterative asymmetric division of stomatal precursor cells (Bergmann & Sack, 2007; Geisler et al., 2000; Larkin et al., 1997). Impinging on one of these processes often results in an alteration in cell number and/or cell size, affecting final leaf size and/or shape and plant biomass (Gonzalez et al., 2012; Nelissen et al., 2016; Vercruysse et al., 2020).

Leaf growth‐regulatory genes encode proteins of diverse functional classes involved in the regulation of a single or multiple cellular processes (Gonzalez et al., 2010; Hepworth & Lenhard, 2014; Krizek, 2009; Schneider et al., 2021). The PEAPOD (PPD)/KINASE‐INDUCIBLE DOMAIN INTERACTING (KIX)/STERILE APETALA (SAP) module is an example of a leaf growth‐regulatory module that is highly conserved to regulate cell number in numerous eudicot species (Schneider et al., 2021). Upon down‐regulation of the PPD/KIX complex or up‐regulation of STERILE APETALA (SAP), mediating post‐translational degradation of the PPD/KIX complex, cell division is significantly increased in leaves, resulting in significant shoot biomass increases in up to about 50% (Schneider et al., 2021). Besides the PPD pathway, there are several other key regulators of organ growth with highly conserved functions, such as the CYTOCHROME P450 78A (CYP78A) family (Anastasiou et al., 2007; Stransfeld et al., 2010; Wang et al., 2008), and the CELL NUMBER REGULATOR (CNR) (Guo et al., 2010), TONNEAU1 Recruiting Motif (TRM) (Guo & Simmons, 2011; Wang, Pan, et al., 2019), SUN (Sun et al., 2017), OVATE (Snouffer et al., 2020), YABBY (Strable et al., 2017; Zhang et al., 2019) and WOX (Cho et al., 2013; Wang et al., 2017) proteins. Several of the identified leaf growth regulators also regulate fruit or seed size (Chen et al., 2021; Monforte et al., 2014; Schneider et al., 2021; Sun et al., 2017), suggesting that the growth processes may, at least in part, be similarly regulated in above‐ground organs. Although increasing sink size could be a way to increase yield, an increase in seed size may also result in a concurrent but adverse impact on the harvest index (Masclaux‐Daubresse & Chardon, 2011). For instance, in soybean lines in which the *PPD* orthologue *BIG SEEDS 1* (*BS1*) is down‐regulated, seed size is increased but accompanied with the production of less seeds (Ge et al., 2016). Some growth regulators also connect organ size to other important yield‐related traits. For instance, KLU, a member of the CYP78A family, acts as a positive regulator of organ growth, leaf longevity and drought tolerance in maize plants (Jiang et al., 2021), while GROWTH REGULATING FACTOR 5 (GRF5) stimulates leaf size, photosynthesis and leaf longevity (Vercruyssen et al., 2015). The strong effects on diverse plant organs in numerous species indicate that targeting these conserved key leaf growth‐regulatory pathways (Vercruysse et al., 2020) may offer great potential to increase crop productivity.

Besides cell proliferation, cell expansion also contributes to final leaf size, and a close coordination between both processes is fundamental for proper organ development (Andriankaja et al., 2012; Vercruysse et al., 2020). Leaf cells can loosen or tighten their primary walls, revealing that the molecular processes underlying irreversible cell wall expansion are dynamically controlled. Cell expansion is proposed to be predominantly regulated by EXPANSINs (EXPs), known for a long time to play a crucial role during cell wall loosening (Cosgrove, 2000a, 2000b; Vercruysse et al., 2020) and to integrate various developmental, genetic and environmental growth signals (Muller et al., 2007). Besides EXPs, XYLOGLUCAN ENDOTRANSGLUCOSEYLASE/HYDROLASEs (XTHs), PECTIN METHYLESTERASEs (PMEs) and pectin materials have been identified as key components of cell wall mechanics and therefore growth control (Cosgrove, 2015; Phyo et al., 2017; Schmidt et al., 2016; Vercruysse et al., 2020). The most recent discoveries also point towards a role for cell wall sensor pathways, such as FERONIA (Cheung & Wu, 2011; Li et al., 2016) and THESEUS1 (Cheung & Wu, 2011; Hématy et al., 2007) receptor‐like kinases (RLKs), in response to diverse signals. FERONIA activates the production of ROS, known to be important mediators for diverse processes, including cell expansion and stress resistance (Ji et al., 2020). Although a short list of cell expansion modulators has been established, their exact role on affecting cell wall extensibility is for most unknown, and the underlying molecular mechanisms are underexplored. It is of crucial importance to understand how these molecular actors coordinate the response to environmental stresses, because any growth modification in plant leaves is concurrent with, and possibly controlled by, changes in cell wall properties (Cosgrove, 2018). Particularly relevant will be a better understanding of their link to the water fluxes towards the growing cells (Touati et al., 2015), and therefore also plant growth, survival and stress resistance (Chenu et al., 2009). This indicates that basic mechanisms underlying organ growth may also link towards other processes that might be important for obtaining climate‐resilient crops and a sustainable agriculture.

In eudicots, such as Arabidopsis, leaves are generally round and contain a reticulate venation pattern, whereas leaves of monocot grasses, such as maize, are narrow and long with a parallel‐like venation pattern (Nelson & Dengler, 1997). Despite these distinct leaf shapes, several studies demonstrated that the cellular and molecular mechanisms governing leaf growth in eudicots and monocot grasses are largely conserved (Liu et al., 2009; Nelissen et al., 2016; Peterson et al., 2010; Raissig et al., 2017). In monocot leaves, however, the proliferation, expansion and mature developmental stages are predominantly separated in a spatial manner with dividing cells located near the base of the leaf, followed by expanding and mature cells positioned towards the tip of the leaf (Avramova et al., 2015; Fournier et al., 2005; Nelissen et al., 2016). In addition, whereas stomata are distributed in a random manner in eudicots, solely following the ‘one‐cell spacing rule’, stomata are organised in a linear manner in grass species (Liu et al., 2009; Peterson et al., 2010; Raissig et al., 2017). Accordingly, not all processes translate across species (Gong et al., 2022), for instance, because grass leaves lack meristemoids, the stomatal precursors in eudicot species, the process of meristemoid asymmetric cell division and the proteins regulating this process, are absent in monocot grasses (Gonzalez et al., 2015; Liu et al., 2009; Schneider et al., 2021; Vatén & Bergmann, 2012).

### 4.2 Improving crop phenology

Given the more frequent occurrence of extreme weather conditions, altering developmental stages is also a key factor to adapt the crop life cycle to abiotic stress. Although a longer growing season means more photosynthesis, earlier flowering might be an option to avoid heat stress during the grain‐filling period (Gouache et al., 2012). For example, wheat phenology (number of days between the sowing and heading or flowering time) is regulated by a small number of loci (Bogard et al., 2014; Fischer, 2011; Trevaskis, 2010) and as such gives the opportunity for researchers and breeders to directly use this genetic information to enhance breeding programmes. In brief, wheat phenology is defined by three components (Le Gouis et al., 2012; Rousset et al., 2011): (i) vernalisation, that is the requirement for exposure to cold temperatures to induce the transition between the vegetative and the reproductive phase, is mainly governed by the *Vrn* gene family, including *Vrn‐A1*, *Vrn‐A2* and *Vrn‐B3* on homologous chromosomes 4, 5 and 7; (ii) the photoperiod, that is the sensitivity to the inductive effect of long days on the transition between the vegetative and the reproductive phase, is mainly governed by the *Ppd‐1* genes family located on homologous chromosomes 2, including *Ppd‐D1*, *Ppd‐B1* and *Ppd‐A1*; and (iii) earliness per se, referred to as the remaining variability independent of vernalisation requirement and photoperiod sensitivity, is the less known component with only one locus mapped as a Mendelian factor located on chromosome 1D (*Eps‐D1*).

Increasing yield by altering the flowering time as a means to avoid heat stress during anthesis or grain filling could, however, also have a countereffect, because the duration and therefore total amount of radiation interception is directly linked to crop biomass accumulation (Monteith, 1972). Accordingly, an optimal balance among these processes will be required to optimise crop performance. In parallel, alterations of developmental stages to counteract the effect of abiotic stresses will need to be accompanied by other adaptation strategies, such as improving genetic tolerance against these stresses (Gojon et al., 2022).

### 4.3 Root development

In addition to the biological processes underlying the growth and development of the above‐ground plant parts, the roots deserve discussion in the context of yield potential. Roots are still an under‐appreciated component of crop productivity despite providing the means to capture soil water and essential mineral elements required to generate the canopy that provides photosynthates. In addition, a well‐developed root system allows for an increased crop resilience in periods of water deficit, nutrient deficit and adverse soil conditions such as compaction (Pandey et al., 2021). Roots constitute a substantial proportion of plant biomass but are rarely measured in experiments involving yield components and their link with traits processes determining yield potential is usually not considered. Variation in root growth may represent a source of genetic improvement that could support higher canopy photosynthesis (Murchie & Reynolds, 2013). However, roots have a higher respiratory cost than shoots and form intricate growth‐promoting interactions with microorganisms in the soil (rhizosphere). Root system properties such as architecture (e.g. depth, root front velocity, root angle, seminal root number, root hairs and total root length) could be improved to enhance resource capture, especially under conditions where water, essential microbes or nutrients are (partially) limiting (Manschadi et al., 2006; Ober et al., 2021; Xie et al., 2017). Moreover, there may be signalling links between root processes and photosynthetic function, such as the observation that lowering stomatal density via the gene *EPIDERMAL PATTERNING FACTOR 1* (*EPF1*) can induce root aerenchyma formation (Mohammed et al., 2019).

To summarise, modulation of organ growth offers major potential for increasing plant yield. Various regulators of leaf growth, their targets and interacting proteins as well as the interactions between these growth regulatory modules have been described (Beemster et al., 2005; Gonzalez et al., 2012; Hepworth & Lenhard, 2014; Nelissen et al., 2016; Tsukaya, 2013; Vercruysse et al., 2020). Besides getting a better view on the growth and development of the above‐ground plant parts, root growth, development and architecture will need to be further unravelled in the coming decades. Specifically, a better understanding is required of how root phenotypes might influence other plant traits, such as photosynthesis or nutrient uptake, and vice versa. Root‐to‐shoot ratio is a plastic trait in plants (Ledo et al., 2018). In the past, breeding has unrelentessly favoured above‐ground biomass production, often penalising root systems. For example, this has led to the neglect and even loss of perennial crop plants, mainly cereals (Crews & Cattani, 2018). In an increasingly dry and hot world, investing in a root system that provides sufficient supply of water and nutrients to the above‐ground biomass will also be a useful and complementary strategy to future‐proofing plants (Lombardi et al., 2021).

## 5 CONCLUSIONS AND FUTURE POTENTIAL

To future‐proof agriculture, our current crops will need to be re‐imagined improving their performance. In this review, several major yield components and the underlying molecular mechanisms have been presented that contribute to intrinsic yield potential: photosynthesis, nutrient partitioning and remobilisation, leaf longevity, seed filling and some aspects of plant organ growth and development (Figure 1). Various molecular pathways underlying these biological processes have been identified that offer great potential to increase crop productivity. Manipulating C and N partitioning to enhance yield of harvestable plant organs, for instance, has been the basis of crop domestication and remains a major avenue for increasing not only yield but also stress resilience and nutritional value of seeds (Yadav et al., 2015). Despite this, uncertainties remain, and significant research is needed to address them. Improving the efficiency by which light energy is converted into biomass has, for instance, not yet been a target of direct selection, and the options to improve the light conversion efficiency to increase crop yield are still largely underexplored (Long et al., 2015; Zelitch, 1982).

Food security will require a sustainable increase in crop yields with guaranteed nutritional value. Whilst improving nutritional quality is outside the scope of this review, methods to increase protein, vitamin and nutrient levels can be found in Scharff et al. (2021), and any future crop improvement strategies must consider both yield and nutritional quality. This is of particular importance given that in the context of rising CO_2_, some loss of nutritional value is also expected (Donnelly et al., 1999; Ebi et al., 2021; Myers et al., 2014).

To meet both food and nutritional security, we will need to improve crop nutrient economy whilst simultaneously increasing agricultural production without increasing the use of fertilisers, which pose further pressure on the environment. It is clear that improving the photosynthetic processes, nutrient remobilisation, and growth and development of root and shoot systems can contribute to achieve these goals. So far, these traits have been studied independently, but metabolic pathways are integrated in the organismal physiology of plants. Connections between traits determining crop yield have become more clear, indicating that, although impinging on individual processes offers perspectives to increase crop productivity, the processes underlying crop yield are strongly interlinked and should be considered holistically to develop high‐yielding crops and avoid adverse off‐target effects. Therefore, it will be important not only to extend knowledge of individual pathways, regulators and their contributions to plant performance, but to also analyse how genes, at the network level, cooperate to exert specific functions and to reveal the connections between the different networks and biological processes. For instance, N uptake and use are not only essential determinants of efficient photosynthesis but are also highly interlinked with photorespiration in different tissues and organs, both at the intracellular and intercellular level (Nunes‐Nesi et al., 2010). N uptake and use are also influenced by plant growth and development, for example through plasticity of root structural and transport characteristics that modulate exploration of the soil and intake capacity (Gautrat et al., 2021; De Pessemier et al., 2022). In turn, the capacities of the roots to acquire N depend on C fixation by photosynthesis, with root C availability representing a major constraint affecting root architecture and development (Freixes et al., 2002). These multiple interconnections between N assimilation and C metabolism are of major importance for crop production, and eco‐physiological studies have demonstrated that C and N intake are the major limiting variables in models of plant biomass production (Foulkes et al., 2009).

The challenge in breeding for crop optimisation lies in combining or stacking promising plant traits, requiring a holistic approach that encompasses the manifold processes underlying productivity (Figure 1). This is becoming possible because of improved technical, (field) phenotyping and network engineering capacities (Araus et al., 2018; Juliana et al., 2019; Reynolds et al., 2020), for which crop modelling approaches show high potential (Yin et al., 2022). In addition to increasing crop performances by using modern breeding tools (marker‐assisted selection and/or genomic selection), a recent advance in climate change adaptation is the combined use of crop models and genomic prediction to define cultivar ideotypes (Bogard et al., 2014, 2021; Gouache et al., 2017). Compared to the classical crop modelling approach, defining ideotypes using marker‐based crop model parameters that take into account the genetic structures of phenology and other traits in the available germplasm (e.g. Gu et al., 2014; Kadam et al., 2019) avoids the risk of defining ‘pure in silico’ ideotypes that may be difficult to obtain by breeding or marker‐assisted selection due to genetic limitations, such as linkage drag and pleiotropic effects.

Superior high‐yield crop varieties will need to be harnessed in the context of imminent effects of climate change. Abiotic stresses, such as heat, salinity, water management (e.g. drought, flooding) and freezing, will need to be met with strategies for resistance, resilience and/or acclimation and better resource (e.g. water, phosphorus, N and minerals) uptake and use efficiency (Gojon et al., 2022). Part of the grand challenge to improve crop yields is to combine yield potential with resilience to both biotic and abiotic stressors (Harbinson et al., 2021). Future crops must have good yield stability with a high resilience to adverse climate and volatile weather conditions if we are to minimise the environmental impact of agriculture. Notwithstanding the complexity of the system, some important control points have been identified that could be explored to improve crop productivity. For some processes, optimisation in low‐stress conditions was also shown to increase crop performance under abiotic stress conditions (Nuccio et al., 2015; Voss‐Fels et al., 2019). Alterations of the T6P/SnRK1 pathway, for instance, result in positive changes in photosynthesis, growth and development (Paul et al., 2001, 2020; Pellny et al., 2004) in non‐stressed conditions, as well as with drought stress during the flowering period (Nuccio et al., 2015).

## FUNDING INFORMATION

No funding was received to support this research.

## CONFLICT OF INTEREST

The authors declare that they have no conflict of interest.

## Data Availability

Data sharing is not applicable to this article as no new data were created or analyzed in this study.
